# Repurposing FDA-Approved Drugs Against Potential Drug Targets Involved in Brain Inflammation Contributing to Alzheimer’s Disease

**DOI:** 10.3390/targets2040025

**Published:** 2024-12-04

**Authors:** Catherine Sharo, Jiayu Zhang, Tianhua Zhai, Jingxuan Bao, Andrés Garcia-Epelboim, Elizabeth Mamourian, Li Shen, Zuyi Huang

**Affiliations:** 1 Department of Chemical and Biological Engineering, Villanova University, Villanova, PA 19085, USA; 2 Department of Biostatistics, Epidemiology and Informatics, University of Pennsylvania, Philadelphia, PA, USA

**Keywords:** brain inflammation, Alzheimer’s disease, druggability analysis, drug-like density, single-nuclei sequencing analysis, small molecule inhibitor, drug repurposing, docking

## Abstract

Alzheimer’s disease is a neurodegenerative disease that continues to have a rising number of cases. While extensive research has been conducted in the last few decades, only a few drugs have been approved by the FDA for treatment, and even fewer aim to be curative rather than manage symptoms. There remains an urgent need for understanding disease pathogenesis, as well as identifying new targets for further drug discovery. Alzheimer’s disease (AD) is known to stem from a build-up of amyloid beta (Aβ) plaques as well as tangles of tau proteins. Furthermore, inflammation in the brain is known to arise from the degeneration of tissue and the build-up of insoluble material. Therefore, there is a potential link between the pathology of AD and inflammation in the brain, especially as the disease progresses to later stages where neuronal death and degeneration levels are higher. Proteins that are relevant to both brain inflammation and AD thus make ideal potential targets for therapeutics; however, the proteins need to be evaluated to determine which targets would be ideal for potential drug therapeutic treatments, or ‘druggable’. Druggability analysis was conducted using two structure-based methods (i.e., Drug-Like Density analysis and SiteMap), as well as a sequence-based approach, SPIDER. The most druggable targets were then evaluated using single-nuclei sequencing data for their clinical relevance to inflammation in AD. For each of the top five targets, small molecule docking was used to evaluate which FDA approved drugs were able to bind with the chosen proteins. The top targets included DRD2 (inhibits adenylyl cyclase activity), C9 (binds with C5B8 to form the membrane attack complex), C4b (binds with C2a to form C3 convertase), C5AR1 (GPCR that binds C5a), and GABA-A-R (GPCR involved in inhibiting neurotransmission). Each target had multiple potential inhibitors from the FDA-approved drug list with decent binding infinities. Among these inhibitors, two drugs were found as top inhibitors for more than one protein target. They are C15H14N2O2 and v316 (Paracetamol), used to treat pain/inflammation originally for cataracts and relieve headaches/fever, respectively. These results provide the groundwork for further experimental investigation or clinical trials.

## 1. Introduction

The neurodegenerative disease known as Alzheimer’s leads to progressive memory loss and impaired functions. It associated with damaged neurons in the brain which have been linked to build up of insoluble amyloid beta (Aβ) plaques and tau protein tangles[1]. The presence of these abnormal accumulations may directly correlate to the progression of the disease and the increasing severity of symptoms [2]. It is the severity of these symptoms and the increasing number of cases [3] that generate the demand for therapeutic treatment options. There currently are very few FDA approved drugs for the purpose of either diminishing the symptoms of the disease or actually stopping the progression [2], [4], [5], [6], [7]. Other treatments that had been investigated, such as BACE-1 or PSEN-1 inhibitors proved to not have curative effects and thus were not approved [2], [8]. Therefore, there is still a need to identify new targets for drug therapeutics that aim to cure Alzheimer’s.

One potential source of target candidates may come from investigating inflammation in the brain. Inflammation in the brain arises from the combination of abnormal insoluble materials and degenerating tissues. Thus, the increasing amounts of neuronal death, Aβ plaques, and tau tangles that occur during the progression of AD provide a clear link to inflammation of the brain and AD. It has been proven that the cytokines connected to inflammation are chronically upregulated in the areas where AD has impacted the brain [9]. Originally, it was thought that inflammation arose alongside AD due to dead tissue and abnormal proteins; however, there is the potential that inflammation that arises due to Alzheimer’s may increase the rate of neurodegeneration and attack living neural tissues [9]. There have been previous attempts to target Alzheimer’s through inflammation via the use of non-steroidal anti-inflammatory durgs, but none have been successful so far [2], [10], [11]. Therefore, there is still a need to identify new Alzheimer’s-inflammation targets.

In order to identify these potential targets, the pathways and proteins relevant to both Alzheimer’s and inflammation must be explored. Alzheimer’s-inflammation is impacted by multiple interdependent pathways in the body. However, it has been shown that the dominant pathway is the classical complement pathway [11], [12], [13], [14]. Therefore, the essential genes from this pathway were the basis for this investigation. The search was also expanded to include associate G protein-coupled receptors (GPCRs), as those have been proven to be ideal targets for drug therapeutics, serving as targets for 50–60& of current drugs[15]. However, while all identified potential targets are relevant to both inflammation and Alzheimer’s, not all may actually be able to bind to a therapeutic. Therefore, the binding capabilities needed to be evaluated for each target. The druggability analysis is the most common approach for this.

The druggablity analysis approaches are generally categorized into either structure-based or sequence-based. A protein is considered druggable if a drug molecule can bind to one of its binding pockets and impact its typical function [16]. Drug-Like-Density (DLID) is one of the most popular criteria for structure-based draggability analysis [17]. Molsoft ICM-Pro software is one of the best platforms for identifying pockets where ligands can dock to and measuring their structure features, which can impact the likelihood of ligand docking [18]. For example, a binding pocket must be accessible to a ligand, and the pocket must be large enough to accommodate the ligand. Protein structures used for analysis in ICM-Pro are obtained from the Protein Data Bank (PDB). Given the protein structures, a pocket searching program (i.e., ICM Pocket Finder) determines the locations of the pockets and extracts their structure features (e.g., aromatic). PockDrug is a similar structure-based method focusing on geometry, hydrophobicity, and aromaticity to evaluate target binding pockets [19], [20]. Similar models include Cavity, which assesses pockets based on 3D structure and ranks them by druggability, employing submodels like CavPharmer for pharmacophore features and CorrSite for allosteric sites [21], [22]. Other platforms like ProteinPlus/DoGSiteScorer [23], [24], [25], fpocket [26], and InDeep [27] analyze pockets and predict druggability through features like pocket geometry and protein-protein interactions. JEDI, which stands for Just Exploring Druggability at protein Interfaces, uses molecular dynamics descriptors [28], while P2Rank leverages machine learning to identify ligand-binding sites from known templates [29], [30], [31], [32], [33]. SiteMap, with 86–96% accuracy, offers quantitative binding site analysis by enhancing binding affinity assessments [34]. Dynamics-aware methods, such as TRAPP (i.e., Transient Pockets with Proteins) [35] and BiteNet [36], [37] evaluate transient pockets. Programs, such as Deep-Surf [38], CryptoSite [39], DEPTH [40], GNN_pocket [41], [42], [43], Kalasanty [44], and PocketMiner [45] were developed to predict the location of cryptic binding sites.

While all the aforementioned tools are valuable for druggability analysis, SiteScore was chosen for this work because it incorporates molecular dynamics information and offers a quantitative approach to provide insights into pocket hydrophobicity and volume. [46]There is some debate regarding the idea that sequence-based methods of druggability analysis are inferior to structure-based [47]. However, there are several sequence-based programs that should still be considered. These include DrugMiner [48], GA-Baggin-SVM [49], DrugHybrid_BS [50], XGB-DrugPred [51], Yu’s Method [52], SPIDER[53], QuoteTarget [54], DrugFinder [55], Sun’s Method [56], and Iraji’s Method [57]. Among all these valuable programs, SPIDER was chosen for this work because it demonstrated reliable cross-validation and test results in Shoombuatong and colleagues’ study [58]. Additionally, SPIDER’s accessible webserver interface allows for practical application in large-scale studies.

This study explored several druggability prediction methods, including two structure-based approaches (DLID and SiteScore) and one sequence-based approach (Stacked PredIctor of DruggablE pRoteins - SPIDER). While these methods help cross-validate the most druggable targets, RNA-sequencing data related to inflammation in Alzheimer’s disease, sourced from databases such as ssREAD portal, were analyzed to confirm the value of these targets in AD. The most druggable and valuable targets identified for addressing AD-related inflammation were subsequently used in Molsoft ICM, a leading ligand-protein docking program [59], [60], to evaluate the potential binding of pre-FDA-approved drugs to these targets. The identified drug targets, along with their potential inhibitors repurposed from FDA-approved drugs, open new avenues for combating AD-related inflammation.

## 2. Materials and Methods

### 2.1 Structure-based Druggability Analysis

The initial target list was obtained from [61], which provided a detailed literature review of proteins involved in inflammation that contributed to Alzheimer’s disease. The first method evaluated for the structure-based methods was Molsoft ICM PocketFinder program established by Merck to find the “drug-like density” of each protein [62]. The PocketFinder program first identifies the pockets available in the provided protein crystal structure. It then reports the volume, hydrophobicity, buriedness, DLID, and other important metrics for each pocket. Each crystal structure has a unique combination of number of pockets and metric scores that make it more or less viable as a drug therapeutic target. The DLID score for each pocket has been shown to be linked the druggability of the target. Pockets are characterized using three main parameters: volume, hydrophobicity, and buriedness. This allows the program to predict whether the protein is capable of bonding with a ‘drug-like’ molecule. The first step of the program is to generate the pockets for the protein and evaluate what protein chains are important to the pockets. Any cofactors were removed so the structure could be properly isolated. The protein residues that are 3.5 angstroms within any surface point of the pocket are then assigned to the pocket’s ‘shell’. PocketFinder is able to report the volume of each pocket directly, while the buriedness is calculated based on the ratio of the solvent-accessible surface area covered by its shell to the solvent-accessible surface area in isolation. The hydrophobicity is calculated from the fraction of the pocket surface in contact with hydrophobic atoms in the shell of the pocket. The actual DLID score calculation begins with the number of drug-like ligand containing (DLLC) pockets with similar volume, buriedness, and hydrophobicity scores versus the total number of pockets with similar scores to find the density of DLLC pockets. After applying a log-based correction factor, the DLID is calculated, as shown in Equation 1 [63].

(1) DLID=log(DLLCneighbors/totalneighors)+1.71

However, this equation requires the pockets to have information available from the pocket database. If this is not the case, then Equation 2 can be used as a substitute [63].

(2) DLID=-8.70+1.71log(volume)+3.94(buriedness)+2.27(hydrophobicity)

Once the scores for volume, hydrophobicity, buriedness, and DLID were calculated for each pocket, they were evaluated to see which targets scored well. It was determined that a DLID score above the 0.5 benchmark was considered “druggable”, meaning it would be likely to bond with a drug therapeutic [63].

Similar to DLID, the second structure-based approach, SiteMap, provides several key metrics alongside its druggability score [64]. These include the number of ‘site points’, which correlates to the size of the site. SiteMap also reports both the hydrophobicity and hydrophilicity characteristics of the site. The ‘balance’ is the ratio of the hydrophobicity to the hydrophilicity. The average hydrophobicity and hydrophilicity score for a tight-binding site is 1.0, while the average balance score is 1.6. This is due to the fact that binding sites tend to have higher hydrophobicity than hydrophilicity scores. The enclosure score evaluates how open the site is to solvent interaction [65].

The SiteScore of a pocket is based on a weighted sum of several of the properties that are discussed below:

(3) $$SiteScore\;=0.0733\;\sqrt{n}+0.6688e-0.20p$$

where n is the number of site points (capped at 100), e is the enclosure score, and p is the hydrophilic score. It is capped at 1.0 to limit the impact of hydrophilicity in charged and highly polar sites. This score is calibrated so that the average SiteScore for 157 investigated submicromolar sites is 1.0. Thus, a score of greater than 1.0 suggests an ideal binding; however, a SiteScore of 0.80 has been found to accurately distinguish between drug-binding and non-drug-binding sites. SiteScore evaluates the site as it would be available for ligand binding. Therefore, while the SiteScore is important, the Dscore is still essential, as it focuses on binding with drugs [64], [65].

Dscore uses the same properties as SiteScore but with different coefficients, as seen in Equation 4:

(4) $$Dscore=0.094\;\sqrt{n}+0.60\;e-0.324\;p$$

For Dscore, the hydrophilic score is not capped. This is one of the essential factors in distinguishing undruggable targets from druggable ones [65], [66]. The use of different functions for binding-site identification, SiteScore, and for classifying druggability, Dscore, can be justified because they have different characteristics and involve different interactions. For example, the PTP1B phosphate pocket can bind with ligands that have nanomolar, or even subnanomolar affinity [65], [67]. However, these ligands are highly active and have charge structures like that of the natural phosphate substrate, making them not drug-like. SiteMap is capable of recognizing that this site can bind ligands but would not be considered druggable [65].

### 2.2 Sequence-based Methods for Druggability Analysis

SPIDER, or Score Protein Interaction Decoys using Exposed Residues, uses machine-learning to create a knowledge-based score function for protein-protein interaction decoys. It is based on the geometric similarity of interfacial residues between docking and native poses. The computational geometry approach known as Almost-Delaunay tessellation is used, which transforms protein-protein complexes into a residue contact network [68], [69], [70]. The FASTA sequences for each target were uploaded to the SPIDER webpage to obtain the Possibility score of the likelihood that a target could be druggable.

### 2.3 Small Molecule Docking for Repurposing FDA-Approved Drugs

The structure of each protein was loaded into the Molsoft ICM program where the top potential binding pocket was identified. Using this pocket, a Monte Carlo simulation was run to assess every possible binding position with the selected drug. This resulted in a binding score that represented the likelihood of the protein binding to that molecule [17], [61], [71], [72], [73], [74]. The standard was set as a score of −20 kcal/mol, and anything below that indicated binding was likely [62]. The drugs used for this docking simulation were a set of approximately 2,500 FDA-approved drugs.

### 2.4 Upstream RNA-sequencing analysis

The raw FASTQ files are obtained datasets GSE138852 [J1] and GSE147528 [J2], comprising 16 samples from entorhinal cortex brain tissue (8 healthy and 8 AD individuals) and 20 samples from both entorhinal cortex and prefrontal cortex tissues (6 healthy and 14 AD individuals). The datasets were processed using Cell Ranger (version 5.0.0) with default settings, and further mapping raw reads to GRCh38 reference from 10x Genomics. After quantification, genes without detectable counts in any cells were removed, and cells were filtered to include only those with gene and UMI counts failing between the 5^th^ and 95^th^ percentiles, as well as less than 10% of UMIs attributed to mitochondrial genes. For downstream analysis, data from the same brain tissue with different conditions were integrated using both *FindIntegrationAnchors* with canonical correlation analysis method to address batch effects, and *IntegrateData* function. The processed *seurat* object were normalized with a scaling factor of 10,000 and further log-transformed. Highly variable genes were defined using *FindVariableGenes* and *ScaleData* was applied to center gene expression. After quality control, the resulting data are consisted of 82,007 cells with 26,754 genes for data in entorhinal cortex brain tissue, and 94,558 cells with 27,590 genes for data in prefrontal cortex brain tissue. It performed principal component analysis to identify clusters based on significant variability, and *FindClusters* at a resolution of 0.5 revealed 21 clusters in the entorhinal cortex and 17 in the prefrontal cortex respectively.

To categorize these clusters identified for major brain cell types, the following marker genes were used: SLC17A7, SNAP25 for excitatory neurons, GAD1, and GAD2 for inhibitory neurons, SLC1A3 or GFAP, and AQP4 for astrocytes, PECAM1 and VWF for endothelial cells, PTPRC, P2RY12 for microglia cells, PDGFRA, CSPG4 for oligodendrocytes precursor cells, and CLDN11, MBP for oligodendrocytes. Non-linear dimensional reduction (uniform manifold approximation and projection UMAP) calculation is used for visualizing these datasets.

To identifying inflammation-related targets differentially expressed in a cell-type sub-population by comparing control and AD conditions. ‘pseudo-bulk’ samples were generated by summing all cells from the same condition, and further analyzed using *FindMarkers*. Heatmaps were constructed to present the relative gene expression across cell types, where the value of log-transformed normalized fold change for each gene were transformed to z-score within each cluster to perform differential expression. The significance of differentially expressed genes was determined using p-value adjusted by Benjamini-Hochberg method, with less than 0.05 threshold as statistically significance.

## 3. Results

### 3.1 Druggability Analysis of Targets via Drug-Like Density

For the drug targets recommended by the literature [61] for their involvement in inflammation in Alzheimer’s disease, the results for the DLID scores are summarized below in Table 1. All of the targets with viable structures had at least one pocket that returned a score, except for CD59. While there was a protein structure available, the ICM *PocketFinder* program did not identify any pockets in CD59 that could bind to potential drugs. The majority of pockets that were identified did not have DLID scores that met the druggability threshold of 0.5. The target with the highest number of viable pockets was C5 with 11 pockets, followed by GABA-A-R with 9. C3 had 6, while C4B, RAB7, and C9 each had 5. DRD2 had two pockets. The following targets each had one pocket above 0.5: C1S, VTN, and C1QB. The remaining targets each had identifiable pockets but did not score high enough, due to a combination of poor volume, hydrophobicity, and buriedness scores. However, it is important to note that this did not mean that there would be no drugs that could bind to these molecules. Having a low druggability score meant that the chances were lower than those that did reach the 0.5 threshold. This was ultimately reflected when the small molecule docking program was run, as those targets were able to bind some of the FDA-approved drugs, but much less than those with higher DLID scores. The results from both the *PocketFinder* run and the DLID assessment are summarized in Table 1.

**Figure 1.**
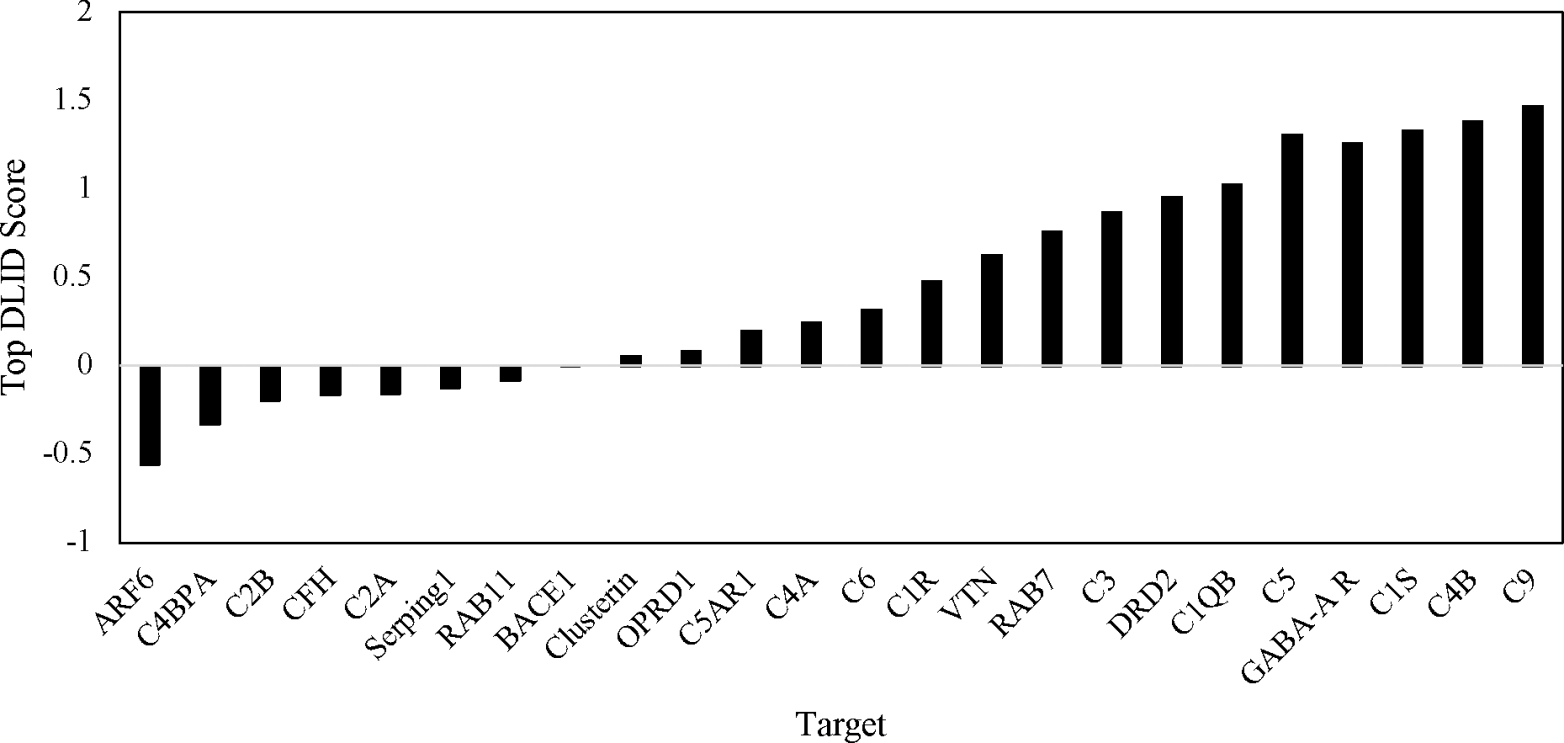
The DLID score returned by the ICM PocketFinder program to evalute the druggability of each target.

The highest DLID score for each target is shown in Figure 1. The trend indicated that having the highest number of pockets did not correlate to having the top-scoring pockets. C5, which had the greatest number of pockets, only had the fifth-highest DLID score. C9 ultimately had the highest singular pocket score, followed by C4B, C1S, and GABA-A R. ARF6 had the lowest DLID score. Seven of the targets had top scores in the negatives, with another seven having positive scores that were just below the 0.5 threshold.

### 3.2 Druggability Analysis of Targets via SITEMAP

**Figure 2.**
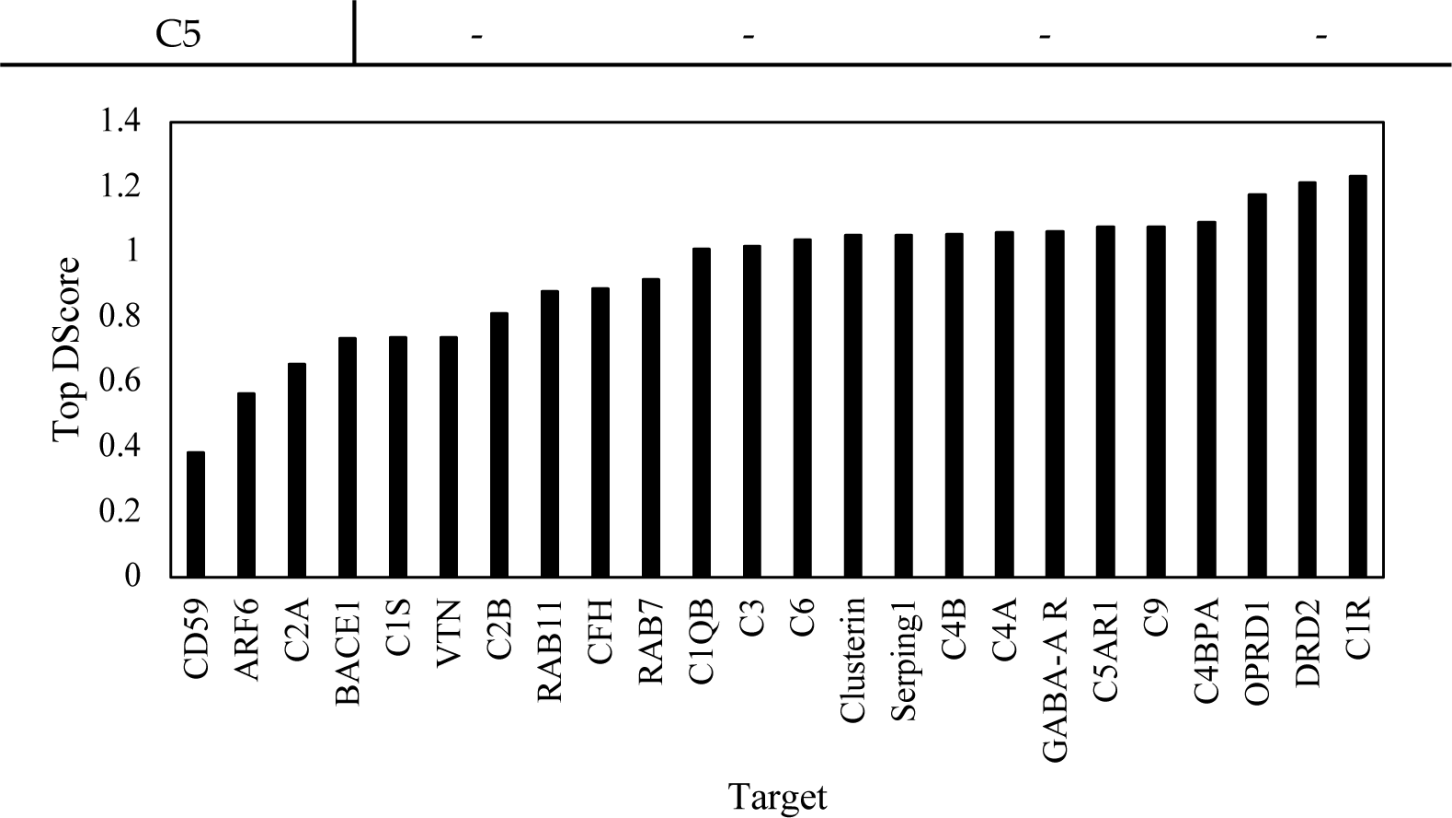
Top DScore returned by the SITEMAP to evaluate the druggability of each target.

Although SiteMap reported both the SiteScore and DScore to reflect the binding ability of the top pockets, the DScore was specifically calibrated to reflect the druggability of the pocket. The results from the SiteMap analysis were thus analyzed around the DScore in order to compare it to the other programs. The top results of the SiteScore, DScore, volume, and balance are reported below in Table 2. C5 did not have any pockets with SiteScores that met the threshold to be reported by the program. However, unlike the DLID scores, CD59 did have one pocket that met the threshold. Additionally, SiteMap reported the top five pockets for each target if enough pockets met the criteria. C4A, C2B, BACE1, RAB11, ARF6, VTN, and CD59 each had less than 5 viable pockets. This generally matched the trends from the DLID results, as the majority of those targets did not have high-scoring pockets for the DLID scores either. The highest DScore result for each target is depicted in Figure 2.

### 3.3 Druggability Analysis of Targets via SPIDER

**Figure 3.**
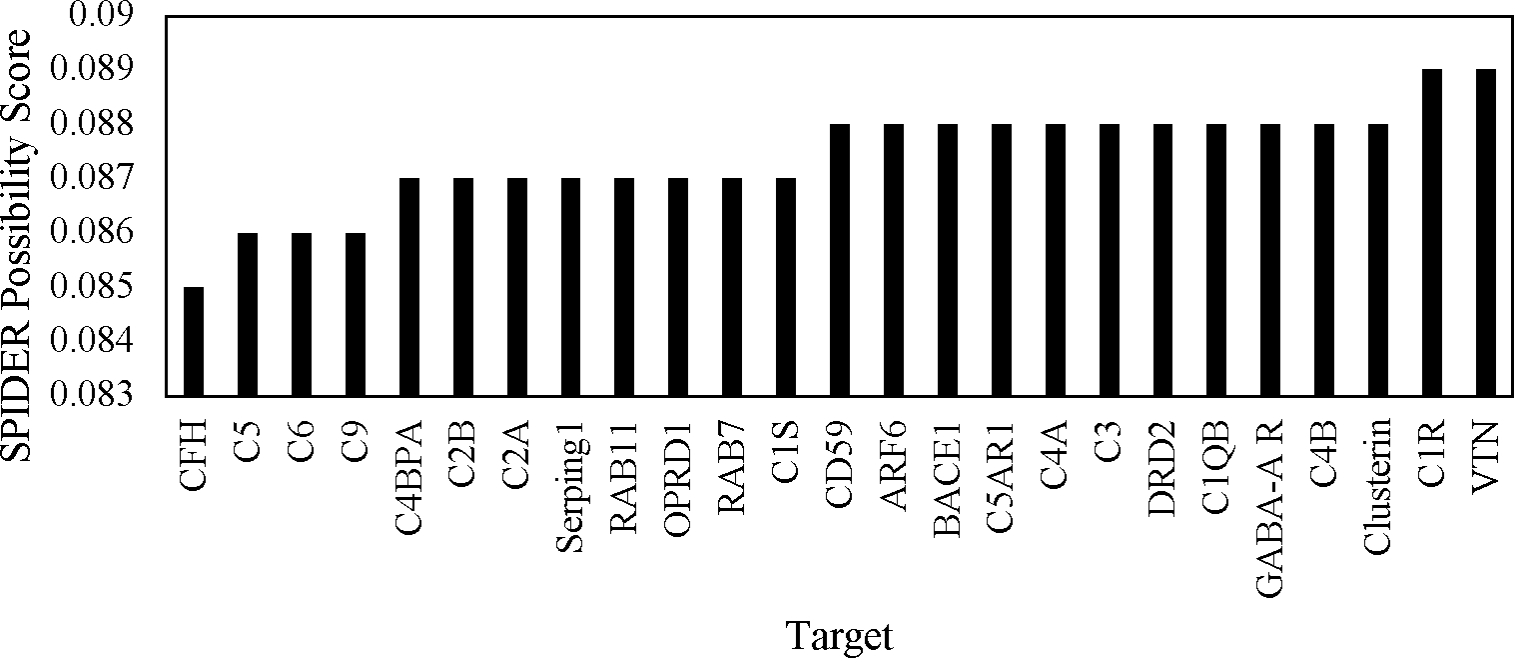
Possibility scores returned by SPIDER to evaluate the druggability of each target.

Once each FASTA file was uploaded to SPIDER, the Possibility score was reported for each target. All of the scores fell between 0.085 and 0.089 as shown in Figure 3. CFH had the lowest score at 0.085, while C1R and VTN both had the highest score of 0.089. The SPIDER results did not match the trends of DLID and SiteMap scores. This was seen in targets such as ARF6, which was one of the lowest scoring molecules for both DLID and SiteMap, but returned the second highest score in SPIDER. Conversely, C9 was a top-scoring target for the two structure-based programs but received the second-lowest score from SPIDER. However, this likely stemmed from the fact that the results do not have enough variation to reflect major trends. All but six of the targets scored either 0.087 or 0.088. Additionally, CD59, which scored low for SiteMap and was undetectable for DLID, scored towards the high end at 0.088, indicating this program may not be one of the ideal means of evaluating this set of targets.

### 3.4 Comparison of Druggability Analysis Results

In order to effectively compare the druggability results across the three platforms, the data had to be normalized. Each of the methods resulted in a different range of scores that meant trends could not be determined by comparing the original numerical results. The following formula was used to normalize each set of results:

(5) $$x_{\mathrm{new}}=\frac{x_{\mathrm{original}}-\mathrm{minimum}}{\mathrm{maximum}-\mathrm{minimum}}$$

**Figure 4.**
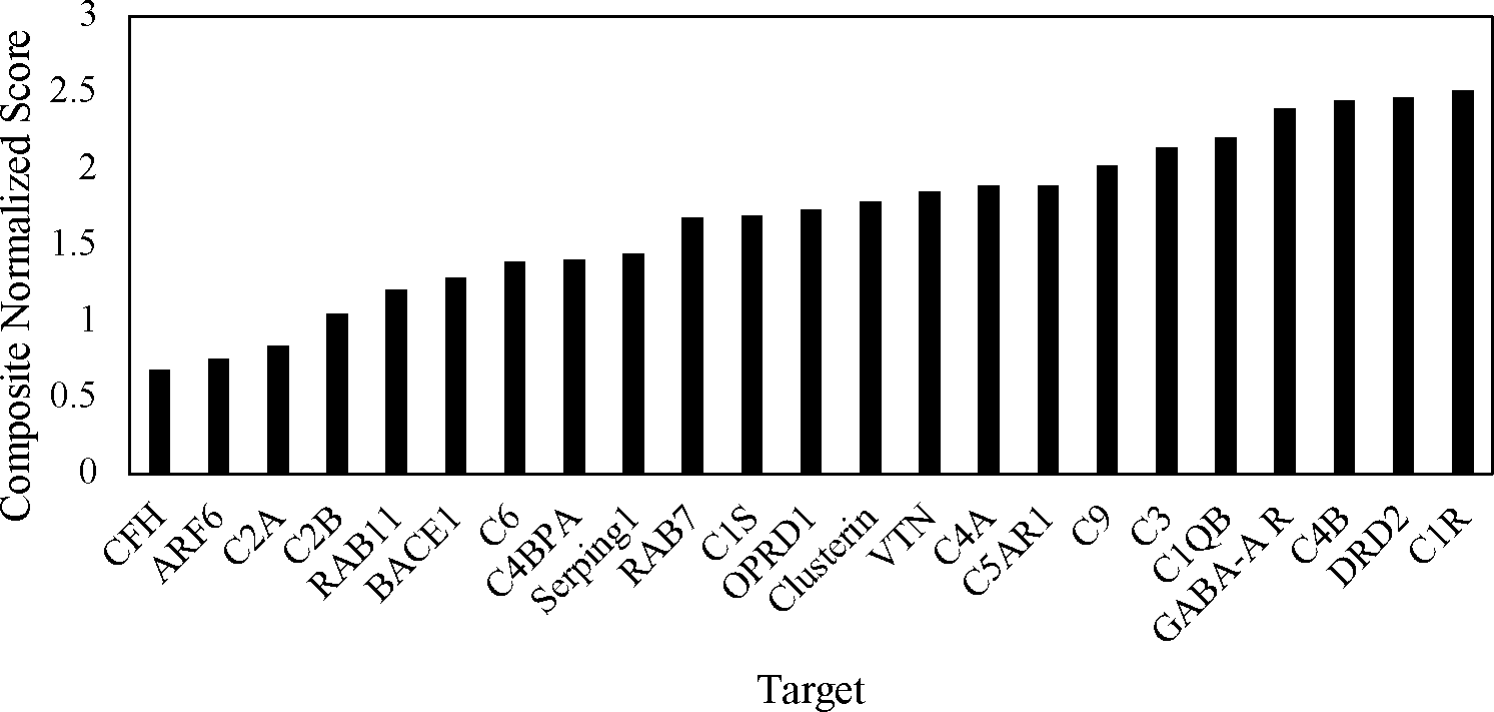
Ranking of targets by combining the normalized results from all three approaches, i.e., the DLID approach, the Spider approach, and the SiteMap approach.

This resulted in three new scores for each target. The two targets that were missing a score from one of the methods (C5 and CD59) were removed from the set since they could not effectively be included. Each target’s 3 scores were then added together to get a composite score that could be used for comparison. The three normalized scores and the composite score for each target are reported in Table 3. The targets were then ranked using the composite score, as shown in Figure 4. The top five molecules in ascending order were C1QB, GABA-A R, C4B, DRD2, and C1R. However, these top five were not all ideal for the small molecule docking that would be performed next. Both C1QB and C1R are very early on in the classical complement pathway. This makes them non-ideal targets for inhibition, as that could have an overly negative impact on the cascade started by those molecules that would affect essential functions in the brain. In moving down the ranking of the targets, C3 was then next but had similar concerns. Therefore, the next two targets, C9 and C5AR1, were selected for the small molecule docking.

### 3.5 Validation of Certain Targes Identified from Druggability Analysis by Single-nuclei Sequencing Data for Inflammation Contributing to Alzheimer’s Disease

**Figure 5,:**
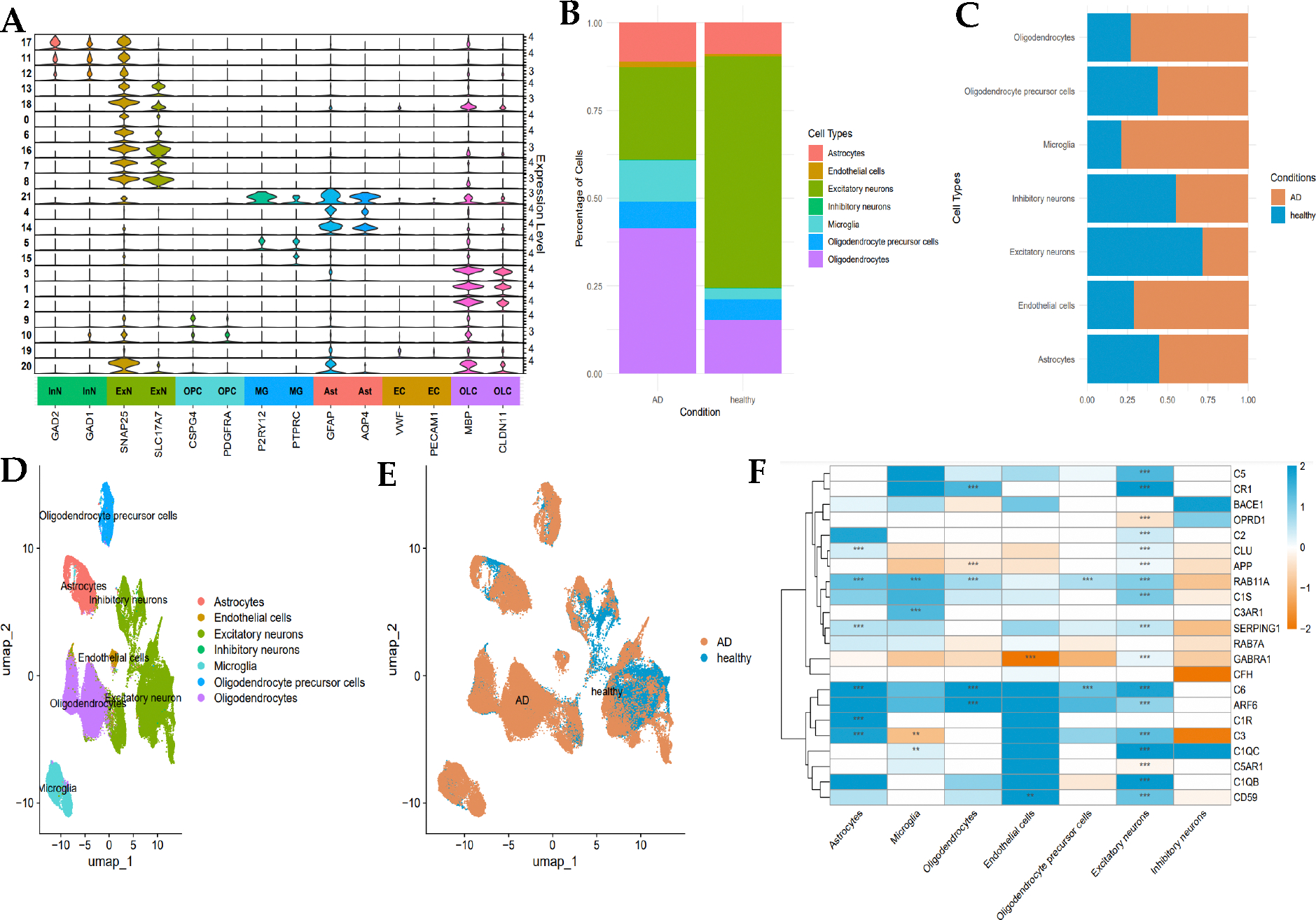
Single-nuclei sequencing data GSE138852 of human entorhinal cortex characterize identified cell types and differential inflammation target expression in ADand healthy brain samples. (A) Stacked violin plot for marker gene expression of seven cell types, including inhibitory neurons, excitatory neurons, oligodendrocyte precursor cells, microglia, astrocytes, endothelial cells, and oligodendrocytes. (B, C) Stacked bar plot representing the cell type distribution in AD and health condition, and cell type proportions between two conditions. (D, E) UMAP visualization for cell distribution of both cell types and different conditions (AD versus Healthy) (F)Heatmap of inflammation targets expression across cell types, and color gradient indicating the expression level (blue for upregulation, and orange for downregulation) with statistical significance (* p-value < 0.05, ** p-value < 0.01, *** p-value < 0.001)

**Figure 6,:**
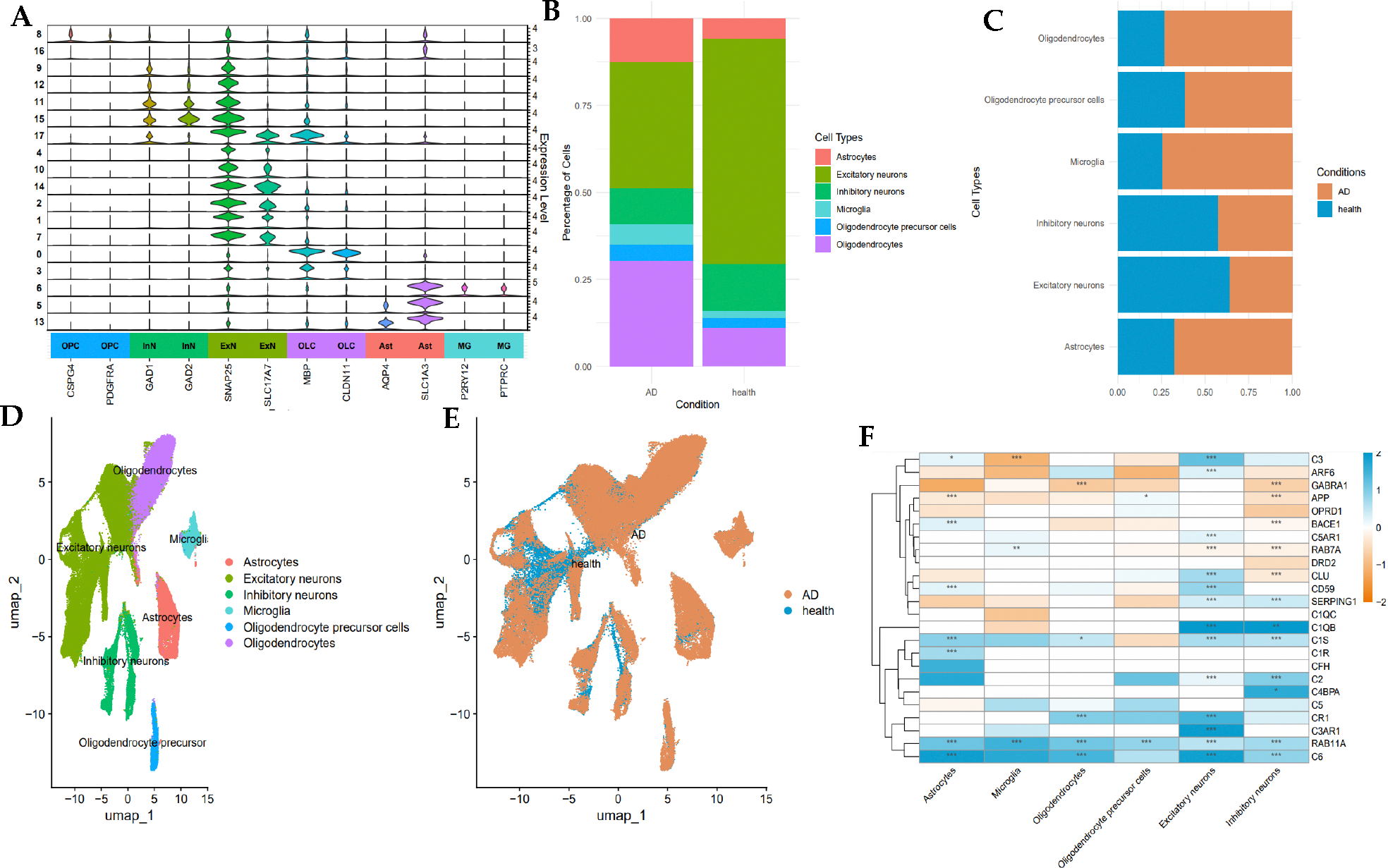
Single-nuclei sequencing data GSE147528 of human prefrontal cortex characterize identified cell types and differential inflammation target expression in AD and healthy brain samples. (A) Stacked violin plot for marker gene expression of seven cell types, including inhibitory neurons, excitatory neurons, oligodendrocyte precursor cells, microglia, astrocytes, and oligodendrocytes. (B, C) Stacked bar plot representing the cell type distribution in AD and health condition, and cell type proportions between two conditions. (D, E) UMAP visualization for cell distribution of both cell types and different conditions (AD versus Healthy) (F)Heatmap of inflammation targets expression across cell types, and color gradient indicating the expression level (blue for upregulation, and orange for downregulation) with statistical significance (* p-value < 0.05, ** p-value < 0.01, *** p-value < 0.001)

To investigate the expression of protein targets involved in inflammation at mRNA level in AD, single-nuclei RNA-sequencing data from 10x Genomic platform available on ssREAD portal, i.e., GSE138852 and GSE147528. This dataset comprised 26 samples from the entorhinal cortex and 10 from the prefrontal cortex, collected from 8 AD and 4 healthy individuals aged 50–91. After quality control filtering, 81,994 cells from the entorhinal cortex (26,754 genes) and 94,558 cells from the prefrontal cortex (27,590 genes) were retained (Figures 5B, 5C, 6B, and 6C). Visualization of single nuclei transcriptomes in uniform manifold approximation and projection (UMAP) space revealed a clear separation into 7 cell types from entorhinal cortex (astrocytes, microglia, oligodendrocytes, endothelial cells, oligodendrocytes precursor cells (OPC), excitatory and inhibitory neurons) (Figure 5D), and 6 from prefrontal cortex (astrocytes, microglia, oligodendrocytes, OPC, excitatory and inhibitory neurons) (Figure 6D).

Differential analysis identified notable differences in cell-type-specific gene expression between control and AD samples, revealing critical patterns that align with disease pathology (Figures 5E & 6E). Endothelial cells in AD exhibited downregulation of the GABRA1 gene, suggesting altered GABA signaling, a pathway known to regulate neuronal activity and implicated in AD pathology [75]. Additionally, neuron cells including inhibitory neuron, and excitatory neuron in Alzheimer’s disease displayed downregulated GABRA1 expression and upregulated CD59, a complement regulatory protein, indicating increased complement activation [76].

Astrocytes in AD showed upregulated expression of subcomponent of Complement Component 1 (C1R, C1S, and C1QB), Complement Component 3, Complement Component 6, and the complement cytolysis inhibitor (CLU) in both entorhinal and prefrontal cortices, highlighting the involvement of complement system in neuroinflammation. This finding aligns with the role of complement system in neuroinflammation, synaptic pruning, and cognitive decline in AD. C1QB is able to complement cascade activation, contributing to neuronal damage, and C1r and C1s form a complex with C1QB, triggering complement activation. C3 plays a role in mediating neuroinflammation attracts astrocytes to amyloid plaques, while C6 participate in membrane attack complex (MAC) formation [77]. The role of CLU is promoting Aβ aggregation reinforces its significance in AD pathology. Similarly, both oligodendrocytes and OPCs demonstrated increased expression of complement proteins which are CR1 and C6. These findings are consistent with the known role of complement activation in synaptic pruning and neuronal damage in AD. Consistently, upregulation of inflammation-related genes (CR1, C2, CLU, C1S, SERPING1, C6, ARF6, C3, C5AR1, C1QB, CD59) also observed across neurons in AD. Moreover, the RAB11A gene, involved in recycling amyloid species, was upregulated across multiple cell types in AD except for endothelial cells, emphasizing its potential role in amyloid pathology [78]. These results collectively indicate that the complement pathway and GABA signaling are disrupted in AD, contributing to inflammation and neuronal injury. The identification of these differentially expressed genes supports the hypothesis that targeting inflammation could be a promising therapeutic strategy for AD.

### 3.4 Small Molecule Docking for Drug-repurposing for the Targets with Highest Druggability

Among the top targets identified from druggability analysis, four of them (i.e., DRD2, C4B, GABA-A-R, C5AR1) were shown in the single-nuclei sequencing data because of their clinical relevance to inflammation involved in Alzheimer’s disease. This validated that clinical values of these targets. The computational docking program were then further implemented below to identify potential FDA-approved drugs as the inhibitors for these targets.

#### 3.4.1 Repurposing FDA-approved drugs to inhibit DRD2

Despite having the highest druggability score of the examined targets, DRD2 had the least amount of small molecule inhibitors that reached the −20 kcal/mol threshold at 8 total molecules. The top scoring molecule was C_20_H_15_F_3_N_4_O_3_, also known as Trovafloxacin or Trovan. Trovafloxacin is a broad-spectrum antibiotic that works by inhibiting the uncoiling of bacterial DNA that is supercoiled through the blocking of DNA gyrase and topoisomerase IV [79], [80]. However, Trovafloxacin was recently taken off of the market because it was shown to increase risk of liver failure[79], [81]. Therefore, the next option would be C_23_H_22_ClN_5_O_3_, also known as Betrixaban, which is an anti-coagulant that works by inhibiting factor Xa. It is used for patients at risk for venous thromboembolism [82], [83]. DRD2 shared two common inhibitors overall, though neither of them was in the top 10 molecules for the other targets. v1099 met the threshold for C9 and is also known as Favipiravir, an antiviral agent used to treat influenza [84]. The other molecule was C_23_H_22_ClN_5_O_3_ (Betrixaban) which was below −20 kcal/mol for C5AR1. Table 4 summarizes the eight inhibitors resulting from the binding simulation with the protein DRD2.

#### 3.4.2 Repurposing FDA-approved drugs to inhibit C4B

C4b had 10 total results that scored below −20, the second least amount of all of the targets. The top-scoring molecule for C4b was C_21_H_18_F_3_N_3_O_5_, also known as Bictegravir. It scored −26.13 kcal/mol. It is a second-generation integrase strand transfer inhibitor that has been approved for the treatment of HIV-1 [85]. C4b had 3 molecules in common with the other targets. The first was m, or Sulfasalazine (C_14_H_14_N_4_O_5_S), which was shared with C9 and C5AR1 but not in the top 10 for either of those targets. It is an NSAID used to treat several diseases with chronic inflammation [86]. The second was v316, which was a top molecule for C9. v316 is also known as paracetmol, which is an alternate form of acetaminophen, commonly known as Tylenol [87]. It is a cyclooxygenase inhibitor that can reduce pain and fever [87], whose exact mechanisms are controversial but likely targets the central nervous system [88]. The last molecule was v487, which met the threshold for C9 and GABA-A-R. However, information on this molecule was lacking. Table 5 summarizes the results from the docking simulation for protein C4b.

#### 3.4.3 Repurposing FDA-approved drugs to inhibit GABA-A-R

GABA-A-R had 28 molecules that scored below −20 kcal/mol, with the top 10 represented below (Table 6). The top scoring molecule was v555, which was not identifiable beyond the fact that it was a covalent organic polymer [89]. GABA-A-R shared eight total inhibitors, all of which were shared with C5AR1 and C9, except for v487 which was shared with C4b as well. These included C_21_H_19_ClN_4_O_4_, v963, v855, v951, C_17_H_15_N_3_O_6_, v461, and C_11_H_6_ClN_3_O_6_.

#### 3.4.4 Repurposing FDA-approved drugs to inhibit C9

C9 had a large number of molecules that scored below −20 kcal/mol with a total of 89. Due to the space constraint, only the top 10 potential inhibitors were shown in Table 7. The top-scoring molecule was v174 at −33.2. v174 is also known as asparaginate, a conjugate base of asparagine [90]. Asparagine is essential to the metabolic control of cell functions in nerve and brain tissue [91]. C9 had 28 inhibitors in common with the other targets.

#### 3.4.5 Repurposing FDA-approved drugs to inhibit C5AR1

C5AR1 had not only the largest number of molecules that scored below −20 kcal/mol, but also the best-scoring molecule. This target had 131 molecules with acceptable scores, with the top one being C_10_H_9_N_5_O at −35.3 kcal/mol (Table 8). This molecule is also known kinetin, which is a proven anti-aging agent [92]. C5AR1 had 29 common inhibitors with the other top targets.

## 4. Discussion

### 4.1 Comprehensive druggability assessment of inflammation-related Alzheimer’s disease targets

The druggability analysis of inflammation-related AD targets using three programs (i.e., DLID, SiteMap, and SPIDER), revealed varying degrees of druggability across the targets. The DLID analysis identified druggable pockets in most targets, but certain targets did not meet the druggability threshold, with CD59 being particularly undruggable as no pockets were identified. Targets like C5, GABA-A R, and C9 had higher numbers of viable pockets, while some had lower scores due to unfavorable pocket characteristics. The SiteMap analysis confirmed the druggability of certain targets, with pockets in C5 and C9 scoring highly, although CD59 had a pocket that met the threshold, unlike in DLID. The SPIDER scores, however, showed minimal variation across targets. This suggested it might not be sensitive enough to reflect major druggability trends. Normalizing the results from all three platforms provided a clearer ranking, with targets like DRD2, C4B, GABA-A-R, C5AR1 emerging as top candidates for small molecule docking. Although some targets such as C1R and C1QB ranked highly, they were deemed unsuitable for inhibition due to their roles in essential biological pathways. The consistency between DLID and SiteMap in identifying druggable pockets in targets like C9 and GABA-A R supports the validity of our results, as different methods with distinct algorithms pointed to similar conclusions. While SPIDER showed less variability, its findings still aligned with other platforms for most high-ranking targets, offering an additional layer of cross-validation. These variations across approaches make sense because DLID focuses on pocket properties like hydrophobicity, while SiteMap evaluates structural features more holistically, and SPIDER emphasizes protein sequences

### 4.2 Drug repurposing for the top drug targets related to inflammation for Alzheimer’s disease

Comparing the top 10 highest binding potential inhibitors across targets shown in Tables 4–8 revealed two contenders: v316 and C_15_H_14_N_2_O_2_. v316 is used to reduce headaches and fever, while C15H14N2O2 is used to reduce pain and inflammation for cataract surgery. showed that only two small molecule inhibitors appeared in the top 10 docking results for more than one target. The first molecule was v316, which was a top result for C4B and C9. This molecule treats pain and fever [87]. The second molecule was C_15_H_14_N_2_O_2_, which was a top result for C5AR1 and C9. This molecule treats pain and inflammation associated with cataracts [93]. It inhibits COX1 and COX2 activity [94]. DRD2 and GABA-A-R did not have any top molecules overlapping with the other targets. However, outside of the top 10, there was significantly more overlap. C4B had 3 of its 10 results overlap with the others. C5AR1 had 29 of its 131 results overlap, while 28 of the 89 C9 results overlapped. 8 of the 28 GABA-A-R were present in the results for the other targets. DRD2 had 2 out of 8 in common with the other targets.

### 4.3 The role of the complement cascade in Alzheimer’s disease progression

While this work focused on protein targets suggested by the literature [61], our approach can be applied to explore additional targets in the future. The involvement of the studied targets in AD-related inflammation was validated through our sequencing data analysis. Further enrichment analysis confirmed that targets such as C1QC, C1QB, CFH, C4BPA, CR1, C9, C6, C4A, CLU, C5, CD59, SERPING1, C1S, C1R, C3AR1, C3, and C5AR1 play a critical role in the complement cascade, which is essential for neuronal development and plasticity [95]. Specifically, complement proteins C1q, C3, and C4 showed increased levels in AD patients brain tissues comparing with healthy people [96]. The mRNA levels of C3 and C4 was observed elevated in the temporal cortex of AD and the increased protein of C3b and the products of the terminal MAC (C5b-C9) in AD brain tissues has also been reported, indicating that that MAC can potentially cause neuronal loss and neurodegeneration in AD [97]. This study suggested significant difference in the mRNA level of C1QC, C1QB, C4BPA, CR1, C9, C6, C4A, CLU, CD59, SERPING1, C1S, C1R, C3AR1, C3 and C5AR1 among AD patients when compared with healthy people through single cell RNA-seq analysis. Complement proteins C1q, C3, and C4 were detected in amyloid plaques and co-localize with neurofibrillary tangles in the brains of AD individuals [98]. Complement proteins C1q, C3b, C3c, and C3d are linked to amyloid plaques and the associated dystrophic neurites during AD progress. Complement C3/C3b has become a crucial factor in synapse elimination by tagging weak synapses and serving as a “eat me” signal. Microglia activated by amyloid-β, and tau protein exhibit morphological alterations and expression of complement receptors CR1, CR3, C3aR, and C5aR1 and secrete a range of immune components, including complement proteins like C1q. The activated microglia having CR3 receptor could identify the C3-tagged synapses and remove them through phagocytosis [99]. Furthermore, the binding of these complement protein to their corresponding receptors triggers inflammation and cytokine secretion. C1q released by activated microglia stimulates A1-reactive astrocytes. A1 astrocytes exhibit elevated expression and release of C3. C3 produced from A1-reactive astrocytes accumulates on amyloid plaques, diminishes synapses, and contributes to neurofibrillary tangles [100]. Additionally, complement C3 has been suggested as an astroglia initiator of nuclear factor-κB (NFκB) signaling via neuronal C3aR, a G-protein-coupled receptor present in various cell types [101]. The NFκB/C3/C3aR signaling pathway induces dendritic and synaptic degeneration in neurons; hence, it is logical that APP/PS1 animals treated with a C3aR antagonist demonstrate enhanced cognitive function [101]. Comprehending the interaction between the complement system and AD is essential for elucidating the molecular mechanisms underlying AD. This work repurposed FDA-approved drugs to regulate druggable components from the complement system for the advancement of targeted therapy strategies.

### 4.4 Limitation and Future Work

Since the X-ray crystal structures from PDB were available for all protein targets, those structures were used for structure-based druggability analysis in this work to ensure the consistency across our selected targets. Although cryo-electron microscopy (cryo-EM) is valuable for visualizing large and flexible proteins, corresponding cryo-EM structures were not available for all target proteins in the PDB. This limitation restricted the use of cryo-EM structures in this work. In addition, certain available cryo-EM structures for the proteins of interest have resolutions worse than the X-ray structures. In future work, we may consider incorporating cryo-EM structures as they become available and achieve higher resolutions that allow us to evaluate any structural differences that may influence druggability results across methods.

Early-stage proteins C1QB, C1R, and C3 were not included for further small molecule docking analysis, because targeting these initial components in the complement pathway could lead to broad and unintended immunosuppressive effects. Although early-stage proteins, like G protein-coupled receptors (GPCRs), are often effective drug targets due to their regulatory roles in signaling cascades, there are instances—particularly within the immune system—where early pathway proteins are avoided to prevent widespread effects. Specifically, in the complement system, targeting proteins such as C1QB and C1R may suppress the entire pathway, which may compromise immune function in a non-specific manner. Future studies may explore the potential of early-stage complement proteins as drug targets, but such strategies require careful consideration of potential immune-related side effects.

In this study, certain FDA-approved drugs were identified with similar binding scores for potential repurposing against Alzheimer’s disease (AD) inflammation (as shown in Table 4). The similarity in binding scores arises because these scores are based on seven binding energy terms, including hydrogen bonds, van der Waals interactions, and electrostatic forces, as calculated in Molsoft ICM. As a result, compounds with comparable interactions across these energy terms can yield similar binding scores. These static binding screening serves as a preliminary step to enable rapid assessment of compounds and reducing resource expenditure. Future work will involve detailed molecular dynamics (MD) simulations to assess dynamic binding stability, which allows us to differentiate compounds further based on their interaction profiles over time. This will be followed by experimental validation of the top-performing candidates to confirm efficacy. Overall, our work establishes a foundation for using MD simulations and experimental studies to refine drug repurposing candidates for AD

## 5. Conclusions

Alzheimer’s disease has been proven to have key connections to brain inflammation, meaning that novel targets may be found through the study of essential proteins and pathways relevant to both diseases. However, in order for these proteins to be viable targets, the druggability of the targets had to be analyzed. This first involved a comprehensive review of both structure-based and sequence-based methods in order to find the best programs for analysis. Once these programs were identified, each of the targets was run through the DLID, SiteMap, and SPIDER programs. The top-scoring potential targets were DRD2, C4b, GABA-A-R, C9, and C5AR1. Single-nuclei sequencing data was used to validate their clinical relevance to Alzheimer’ disease inflammation. For these five most druggable targets, the computational ligand-protein docking program was implemented evaluate FDA-approved inhibitors against each selected target to confirm the binding capabilities of the targets. DRD2 had 8 inhibitors that had scores indicating high potential for binding, while C4b had 10, GABA-A-R had 28, C9 has 89, and C5AR1 had 131. Comparing the top 10 highest binding potential inhibitors across targets revealed two contenders: v316 and C_15_H_14_N_2_O_2_. v316 is used to reduce headaches and fever, while C_15_H_14_N_2_O_2_ is used to reduce pain and inflammation for cataract surgery. For future consideration, *in vitro* experiment for druggability should be conducted to determine if these inhibitors will actually affect the protein targets.

## Figures and Tables

**Table 1.** Summary of ICM PocketFinder and DLID results.

Target	Number of Pockets	Largest Volume	Highest Hydro-phobicity	Buriedness Score	Number of Druggable Pockets

C1R	17	386.7	0.682	0.995	0
C3	37	1024	0.88	0.993	6
C1S	11	1761	0.586	0.824	1
C4B	27	879.1	0.7595	0.9128	5
C4A	4	295.1	0.631	0.859	0
CFH	8	467.5	0.566	0.778	0
Serpingl	3	259.5	0.575	0.803	0
C2A	9	334.9	0.63	0.82	0
C2B	2	393.5	0.568	0.87	0
C5AR1	5	348.8	0.771	0.865	0
RAB7	23	1368	0.593	0.884	5
BACE1	2	428.8	0.623	0.833	0
RAB11	4	387.9	0.529	0.805	0
ARF6	3	278.4	0.544	0.834	0
GABA-A R	62	1923	0.893	0.998	9
OPRD1	13	452.4	0.685	0.917	0
VTN	4	779.5	0.728	0.954	1
C1QB	10	542.9	0.595	0.983	1
C6	16	715.4	0.626	0.87	0
Clusterin	24	1035	0.885	0.949	0
C5	108	1686	0.838	0.98	11
C4BPA	11	250.3	0.693	0.816	0
CD59	0	-	-	-	0
C9	23	614.1	0.895	0.996	5
DRD2	8	620.8	0.832	0.882	2

**Table 2.** Summary of the SiteScore, DScore, volume, and balance results for the pocket with the highest DScore by each target.

Target	DScore	SiteScore	Volume	Balance

C1R	1.235	1.134	332.367	6.057
DRD2	1.213	1.103	167.384	12.82
OPRD1	1.176	1.096	1353.135	3.447
C4BPA	1.093	1.016	343	1.479
C9	1.079	1.048	270.284	0.91
C5AR1	1.078	1.032	228.095	5.406
GABAAR	1.065	1.035	2247.679	0.759
C4A	1.061	1.015	370.44	0.906
C4B	1.056	1.015	673.309	0.708
SERPING1	1.053	1.005	286.748	0.7
CLU	1.052	1.007	294.98	0.732
C6	1.038	0.994	822.857	0.473
C3	1.02	1.034	439.726	0.294
C1QB	1.011	1.126	479.171	0.549
RAB7	0.917	0.987	531.65	0.0005
CFH	0.888	0.903	248.332	0.119
RAB11	0.88	0.881	164.983	0.435
C2B	0.812	0.907	230.839	0.08
C1S	0.738	0.947	98.441	0.223
VTN	0.738	0.821	146.118	0.165
BACE1	0.735	0.806	244.559	0.034
C2A	0.656	0.778	200.655	0.081
ARF6	0.564	0.756	86.093	0.087
CD59	0.384	0.51	45.276	0.021
C5	-	-	-	-

**Table 3.** Comparison of druggability analysis by normalized results.

Target	DLID Score	Spider Score	SiteMap Score	Composite

ARF6	0	0.75	0	0.750
C4BPA	0.113	0.5	0.788	1.401
C2B	0.178	0.5	0.370	1.047
CFH	0.196	0	0.483	0.679
C2A	0.198	0.5	0.137	0.835
Serpingl	0.213	0.5	0.729	1.442
RAB11	0.234	0.5	0.471	1.205
BACE1	0.277	0.75	0.255	1.282
Clusterin	0.302	0.75	0.727	1.779
OPRD1	0.317	0.5	0.912	1.729
C5AR1	0.373	0.75	0.766	1.889
C4A	0.396	0.75	0.741	1.887
C6	0.432	0.25	0.706	1.388
C1R	0.512	1	1	2.512
VTN	0.585	1	0.259	1.844
RAB7	0.651	0.5	0.526	1.677
C3	0.705	0.75	0.680	2.135
DRD2	0.746	0.75	0.967	2.464
C1QB	0.783	0.75	0.666	2.199
GABA-A R	0.896	0.75	0.747	2.393
CIS	0.931	0.5	0.259	1.690
C4B	0.959	0.75	0.733	2.442
C9	1	0.25	0.768	2.018

**Table 4.** Small molecule docking results with DRD2.

Molecule	Name	Binding Score
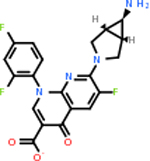	C_2_0H_15_F_3_N_4_O_3_	−24.0
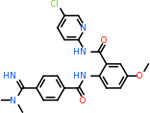	C_23_H_22_ClN_5_O_3_	−23.94
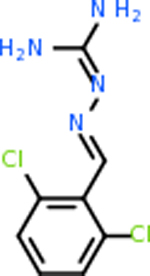	v629	−20.87
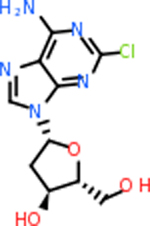	v242	−20.3
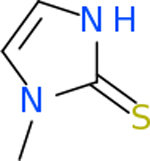	v763	−20.28
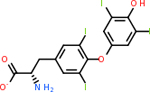	v451	−20.15
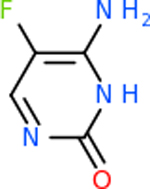	v1099	−20.1
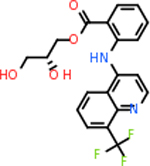	C_20_H_17_F_3_N_2_O_4_	−20.01

**Table 5.** Small molecule docking results for C4b.

Molecule	Name	Binding Score
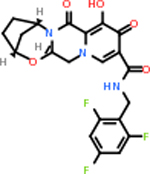	C_21_H_18_F_3_N_3_O_5_	−26.13
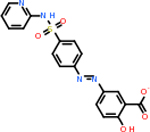	m	−24.36
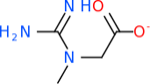	C_4_H_9_N_3_O_2_	−21.65
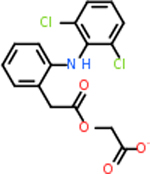	C_16_H_13_Cl_2_	−21.51
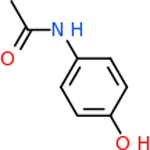	v316	−21.45
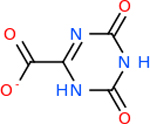	C_4_H_3_N_3_O_4_	−21.21
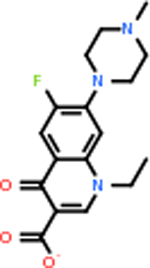	v487	−20.36
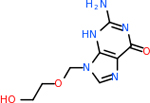	v787	−20.26
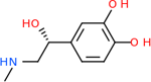	v668	−20.18
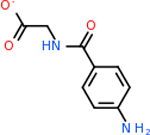	v2148	−20.13

**Table 6.** Small molecule docking for GABA-A-R.

Molecule	Name	Binding Score
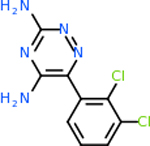	v555	−29.3
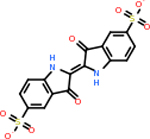	C_16_H_10_N_2_O_8_S_2_	−26.8
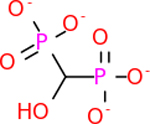	CH_6_O_7_P_2_	−26.4
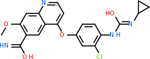	C_21_H_19_ClN_4_O_4_	−25.3
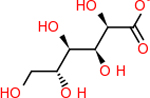	C_6_H_11_KO_7_	−23.8
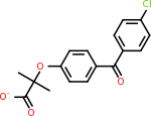	C_17_H_15_ClO_4_	−23.7
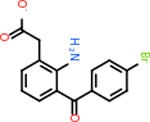	v963	−23.1
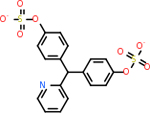	C_18_H_15_NO_8_S_2_	−22.8
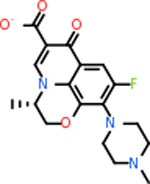	v1165	−22.8
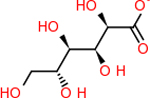	C_12_H_22_MnO_14_	−22.3

**Table 7.** Small molecule docking for C9.

Molecule	Name	Binding Score
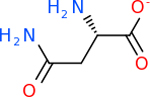	vl74	−33.2
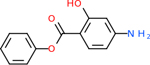	C_13_H_11_NO_3_	−32.7
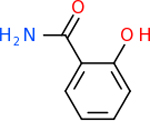	v5147	−31.2
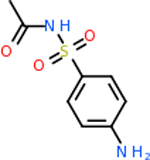	v634	−31.2
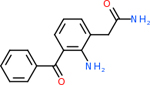	C_15_H_14_N_2_O_2_	−29.2
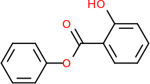	C_13_H_10_O_3_	−29.1
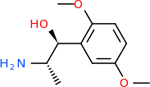	v723	−28.7
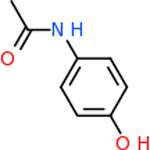	v316	−28.6
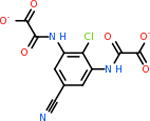	C_11_H_6_ClN_3_O_6_	−28.3
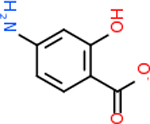	v233	−28.1

**Table 8.** Small molecule docking for C5AR1.

Molecule	Name	Binding Score
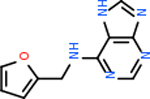	C_10_H_9_N_5_O	−35.3
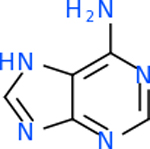	v173	−30.1
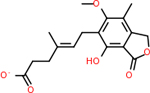	v1024	−29
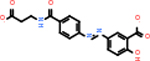	C_17_H_15_N3O_6_	−26.5
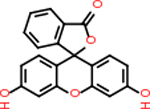	C_20_H_12_O_5_	−26.5
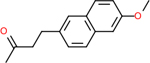	v461	−26.5
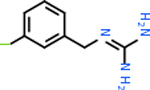	C_8_H_10_IN_3_	−25.9
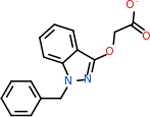	C_16_H_14_N_2_O_3_	−25.8
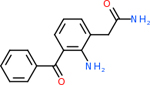	C_15_H_14_N_2_O_2_	−25.7
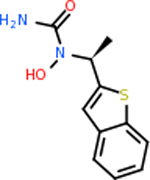	v744	−25.5

## Abbreviations:

AD: Alzheimer’s disease
GPCRs: G protein-coupled receptors
DLID: Drug-Like-Density
PDB: Protein Data Bank
SPIDER: Stacked PredIctor of DruggablE pRoteins
DLLC: drug-like ligand containing
OPC: oligodendrocytes precursor cells
UMAP: uniform manifold approximation and projection
OPC: oligodendrocytes precursor cells
MAC: membrane attack complex
